# Collaborative Visual Analytics: A Health Analytics Approach to Injury Prevention

**DOI:** 10.3390/ijerph14091056

**Published:** 2017-09-12

**Authors:** Samar Al-Hajj, Brian Fisher, Jennifer Smith, Ian Pike

**Affiliations:** 1 Faculty of Health Sciences, American University of Beirut, Beirut 1107 2020, Lebanon; 2 School of Interactive Arts and Technology, Simon Fraser University, Surrey, BC V3T 0A3, Canada; bfisher@sfu.ca; 3 The BC Injury Research and Prevention Unit, BC Children’s Hospital, Vancouver, BC V6H 3V4, Canada; jsmith@bcchr.ca (J.S.); ipike@bcchr.ca (I.P.); 4 Department of Pediatrics, Faculty of Medicine, The University of British Columbia, Vancouver, BC V6H 3V4, Canada

**Keywords:** group analytics, health analytics, human computer interaction, distributed cognition, collaborative visual analytics, problem solving and decision-making

## Abstract

**Background**: Accurate understanding of complex health data is critical in order to deal with wicked health problems and make timely decisions. Wicked problems refer to ill-structured and dynamic problems that combine multidimensional elements, which often preclude the conventional problem solving approach. This pilot study introduces visual analytics (VA) methods to multi-stakeholder decision-making sessions about child injury prevention; **Methods**: Inspired by the Delphi method, we introduced a novel methodology—group analytics (GA). GA was pilot-tested to evaluate the impact of collaborative visual analytics on facilitating problem solving and supporting decision-making. We conducted two GA sessions. Collected data included stakeholders’ observations, audio and video recordings, questionnaires, and follow up interviews. The GA sessions were analyzed using the Joint Activity Theory protocol analysis methods; **Results**: The GA methodology triggered the emergence of ‘*common ground*’ among stakeholders. This *common ground* evolved throughout the sessions to enhance stakeholders’ verbal and non-verbal communication, as well as coordination of joint activities and ultimately collaboration on problem solving and decision-making; **Conclusions**: Understanding complex health data is necessary for informed decisions. Equally important, in this case, is the use of the group analytics methodology to achieve ‘*common ground’* among diverse stakeholders about health data and their implications.

## 1. Introduction

Public health data are ‘data for action’ [1]. They are crucial for the planning, implementation, and evaluation of public health programs, training, and policies. Within the field of public health, health professionals and policy-makers suffer the challenging task of analysing and interpreting heterogeneous and complex health data in order to make time-critical decisions. Dynamic problems, such as those experienced in public health in general, and injury prevention in particular, combine multidimensional elements and constitute what Horst-Rittel called the ‘Wicked Problem’ [2]. We consider injury to be a “wicked problem”, because it involves individual, social, environmental, and policy-related factors. Wicked problems are inherently complex and unstructured problems, characterized by uncertain and incomplete data and they cannot be addressed using well-known problem-solving techniques. Proposed solutions to wicked problems should be judged and assessed based on analysts’ perspectives, assessments, and judgments. As more complex health data is generated and collected, the healthcare community has recognized the need to make sense of these complex, multidimensional data by exploiting information visualization methods and techniques [3]. 

The emerging science of visual analytics seeks to provide theories and empirical methods that can enable application designers to more effectively apply information visualization techniques and computational analysis methods within the context of human decision-making to better deal with complex data [4]. According to Cook and Thomas [4] visual analytics is the ‘science of analytical reasoning facilitated by interactive visual interfaces’. The integration of human capabilities with computational methods is considered fundamental to advancing the analytical process and improving decision-making [5,6]. Visual analytics is a novel approach to addressing complex public health problems that can assist health professionals and policy-makers in resolving problems and making better-informed decisions [7,8,9,10,11].

Visual analytics plays a pivotal role in the data analysis process. It offers analysts two key functionalities: visualization and interactivity. Data visualization amplifies the cognitive capabilities of the analyst, and facilitates problem solving by making the solution to the problem prominent or salient. Interactivity provides a platform to establish an “analytic discourse” [4] between the analysts and the data enabling analysts to ask questions, seek answers, generate hypotheses, and test scenarios. Visualization and interactivity elements can, thus, support analytic discourse between stakeholders and complex data, to enhance the problem solving and decision-making process.

The present research begins with the need to bridge the visual analytics approach and collaborative decision-making methods. Proposed solutions to public health problems must be consistent with a rational analysis of the available health data and take into account the values and beliefs of multiple stakeholders in the relevant community. Thus, communication of thoughts and ideas among stakeholders support the advancement of solutions to complex problems in the context of a structured approach to collaborative decision-making.

This pilot study proposes the design and evaluation of a new methodology termed “group analytics (GA)”, as applied to the complex injury prevention problem in Canada [12,13]. The proposed GA methodology builds upon the Delphi method [14,15,16,17] and advances the paired analytics (PA) methodology [18,19]. The paired analytics methodology is an approach that relies on the collaboration of two analysts, the visual analytics expert (VAE) and the subject matter expert (SME) to reach the intended analytical goal using a specific visualization tool. Group analytics extends the pair analytics methodology to incorporate multiple subject matter experts (SMEs) who interact with each other and with the visual analytics expert (VAE) in a co-located social setting, to solve a given analytical task using a selected visual analysis tool [19,20,21]. The objective of this study is to present the GA method as a way to capture and understand the collaborative visual analysis process among multiple stakeholders and its impact on addressing injury prevention problems and making informed decisions.

## 2. Materials and Methods 

### 2.1. Setting and Participants

Selected local and national injury stakeholders were invited to attend a one-day workshop meeting entitled, “Child and Youth Injury Dashboard using BC CHIRPP data” at BC Children’s Hospital in Vancouver, Canada. A purposeful sampling strategy was employed to select the study participants who were injury stakeholders [22]. The aim of the sampling strategy was to select knowledgeable and experienced injury stakeholders or SMEs. Selection was carried out based on three fundamental criteria: background knowledge (i.e., essential knowledge about the public health injury problem), heterogeneity (i.e., the diversity of skills and expertise to present various perspectives about the injury problem) as well as representativeness (i.e., ensure that SMEs represented constituent peers in their fields within the public health sector). Eight injury stakeholders participated (37% male and 63% female), from various professional backgrounds: public health professionals, health researchers, kinesiologists, epidemiologists, medical practitioners, and policy-makers. 

### 2.2. Data Visualization Tool

**Figure 1:**
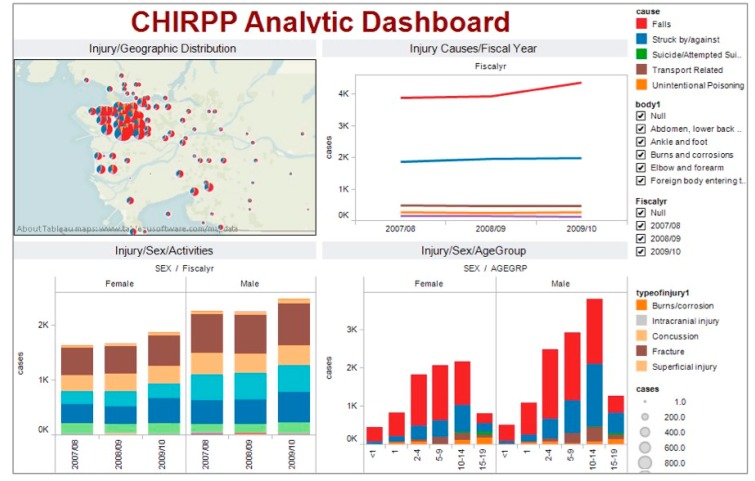
The Interactive Analytical Injury Dashboard (*i*AID) incorporates four visual displays that depict key indicators of the injury problem.

The Interactive Analytical Injury Dashboard (*i*AID) was designed to assist injury stakeholders to understand complex and heterogeneous injury data, and to effectively engage in a real-world injury problem solving process [23] (Figure 1). 

**Figure 2:**
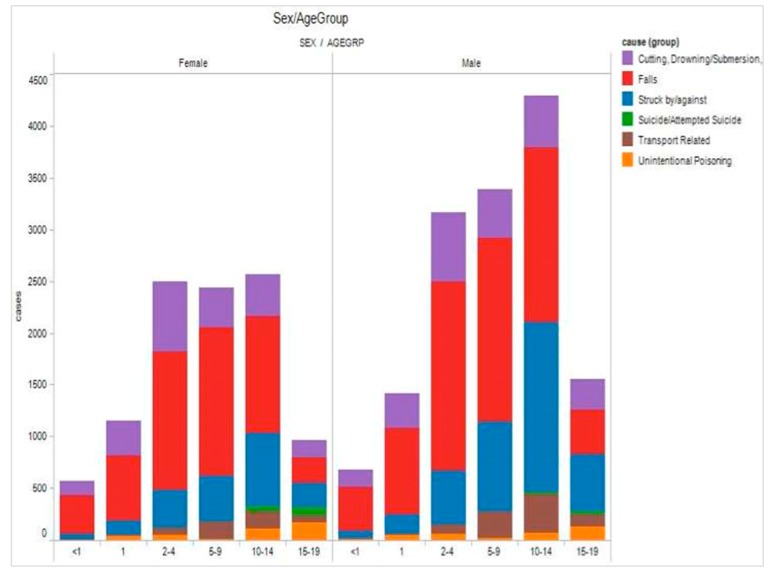
Details-on-demand (sex/age/injury cause).

**Figure 3:**
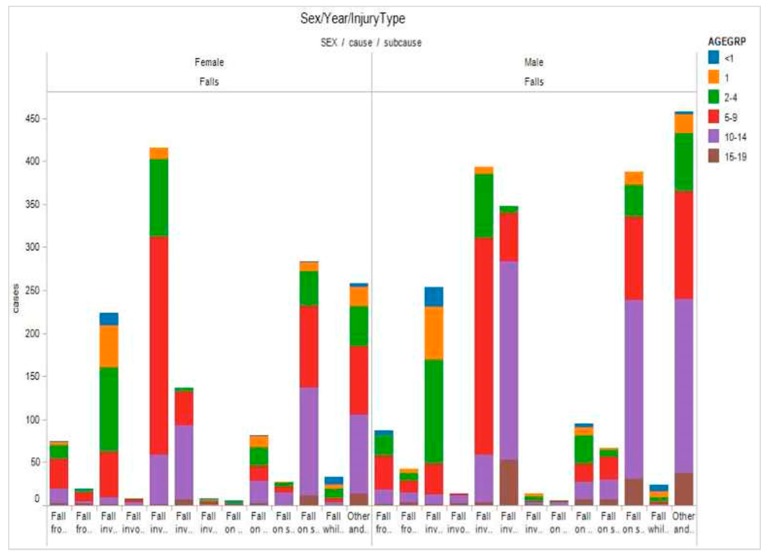
Brushing and linking (sex/injury causes/injury).

Typically, a dashboard is limited to the visual display of key indicators [13]. In addition to key indicators, the *i*AID integrated stakeholders’ analytical process and supported their reasoning through the use of interactive interfaces and customized visualizations in coordinated views [24,25]. The visualizations were selected to depict relevant aspects of the injury problem and to assist SMEs in building knowledge and making informed decisions about dynamic situations for child and youth injury prevention initiatives. Tableau Software (Tableau Software, Seattle, WA, USA) was used to design and build the main visualizations of the *i*AID. Examples are shown in Figure 2 and Figure 3.

The Canadian Hospitals Injury Reporting and Prevention Program (CHIRPP) database was the source of data for this pilot study [12]. CHIRPP is a surveillance system that collects and analyzes data on injured people, mainly children, who are seen at the emergency rooms of 14 participating hospitals in Canada. Prior to the GA sessions, the CHIRPP data was prepared and loaded into the *i*AID dashboard. Further details about CHIRPP data and the *i*AID dashboard are available in Appendix A.

### 2.3. Group Analytics Methodology

During the workshop, the GA methodology was pilot tested in two group sessions. Each session lasted approximately 30-min. The VAE provided SMEs with an overview tutorial of the visualization tool and explained its features and functionalities. A large rectangular table was placed in the middle of the room in order to facilitate each group meeting. An augmented projection mounted on the wall faced all participating SMEs to allow them to synchronously access the interactive information and observe all at once the manipulations of the data when generating customized visualizations. SME interaction data were collected using audio and video recordings, as well as screen capture using Camtasia software (TechSmith Corporation, Okemos, MI, USA) to record on-screen interactions with the visualization dashboard. The research was reviewed and approved by the University of British Columbia and BC Children’s Hospital Research Ethics Board (certificate #H09-01135). 

**Figure 4:**
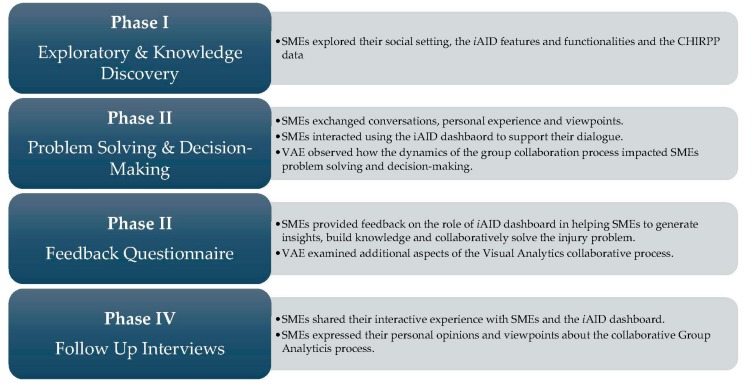
Four phases of the collaborative visual analytics process.

The analytical tasks were carefully designed to simulate real domain tasks, and trigger discussion, argumentation, and collaboration among injury stakeholders. During each GA session, the facilitator presented SMEs with two pre-conceived scenarios. The facilitator invited stakeholders to discuss, share their views and inputs, as well as address each injury scenario with the help of the VAE and the *i*AID. VAE assisted injury stakeholders in manipulating the visual display and customizing the dashboard visualizations based on their needs as they worked through the injury scenarios. The GA methodology pilot testing encompassed four phases (Figure 4). 

### 2.4. Data Collection and Analysis

We used various methods to collect data including ethnographic techniques (e.g., stakeholders’ observations, questionnaires, and follow-up interviews of the focus group). We supplemented these ethnographic techniques with video- and audio-recordings of the GA sessions, as well as screen capturing of the analysts’ interactions with the *i*AID dashboard. These adopted techniques provided detailed and rich descriptions of SMEs collaborative interaction with the tool interface within the context of solving the injury analytical task and making a decision. The collected qualitative data were in the form of field notes, audio- and video-recordings, screen captures, as well as interview notes. 

The analysis of the transcribed audio and video recordings as well as the screen captures of the collaborative sessions were conducted using ATLAS.ti and Chronoviz software (Adam Fouse, University of California, San Diego, CA, USA; ATLAS.ti Scientific Software Development GmbH, Berlin, Germany). Transcripts of the video and audio files were closely examined using open, axial, and selective coding techniques [26] to categorize SMEs recorded verbal and non-verbal interactions and to identify the various themes related to collaborative visual analytics.

The qualitative analysis methodology was grounded in Clark’s Joint Activity Theory (JAT) [27] and the Distributed Cognition (D-Cog) framework [28]. The Joint Activity Theory (JAT) refers to Herbert H. Clark’s theory of “using language”. It is a psycholinguistic theory that adopts language use as a framework to structure time and space coordination of actions between individuals to attend to a common task. The Distributed Cognition (D-Cog) theory, originally developed by Edwin Hutchins, lays the theoretical foundation for coordinating cognitive process among multiple entities including individuals, artefacts (i.e., visualizations), and the environment [28]. Based on JAT, the unit of analysis was specified as ‘any instance of SMEs’ joint activity’ pertaining to the collaborative visual analytics process that occurred throughout the GA session. 

## 3. Results

### 3.1. Common Ground

The analysis revealed evidence of Clark’s notion of ‘common ground’. “Common ground” is defined as the aggregation of “mutual, common, or joint knowledge, beliefs and assumptions” of two individuals [27]. *Common ground* represents joint beliefs and knowledge as the basic foundation upon which individuals’ coordination of actions and collaboration rely. The emergence of three types of *common ground* were classified and documented as follows.

#### 3.1.1. Opening *Common Ground*

Community, cultural, and personal shared bases were emphasized among SMEs and were established based upon their prior shared knowledge and expertise in the field of injury prevention. These shared bases represented the foundation upon which SMEs coordinated their activities to achieve a common purpose and solve the analytic tasks.

SME: *“We had a common goal but we might have taken different pathways to get there, but they’re coming together to talk about it in one group…we have the trauma surgeon, we have the epidemiologist, we have the policy makers, we have the researcher, the coordinator.”*

#### 3.1.2. Cumulative *Common Ground*

New assumptions and shared beliefs were built and accumulated through interactions between SMEs on the *i*AID platform. Insight into the injury data was facilitated by the *i*AID, which SMEs used to establish a common understanding of the injury problem. Interaction between SMEs through verbal communication then generated possible solutions.

SME: *“that exchange (of knowledge) in the group session got us all on the same page.”*

#### 3.1.3. Concluding *Common Ground*

Equity in data understanding led to new beliefs, knowledge, understanding of the injury issue, as well as new approaches shared by SMEs to address the injury problem.

SME 7: *“So, we see that the major thing here is head injuries and eight and four years old and it’s about ‘falls’. So that at least really does start narrowing it down but it still says: ‘So, what are you going to do about that?’”*

### 3.2. Interaction Styles 

**Figure 5:**
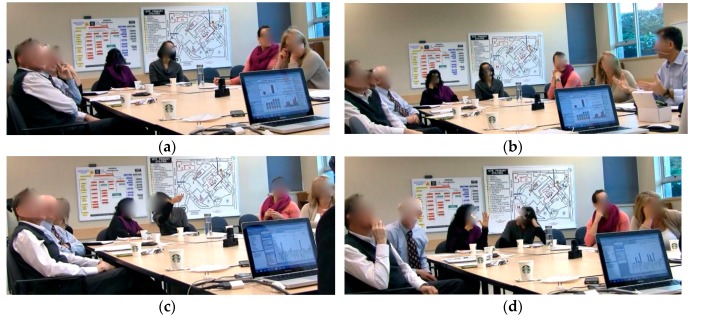
(**a**) All to Artefact (SMEs-*i*AID); (**b**) All to One (SMEs-SME); (**c**) Pairs, rest to Artifact (SME-SME, SMEs-*i*AID); and (**d**) One-on-One (SME-SME).

In both GA sessions, SMEs interacted with other group members by verbally expressing their viewpoints and understanding of the content of the visualizations. The video captured instances of interactions between SMEs (Figure 5). Communications that built and maintained *common ground* while SMEs worked on solving the analytical problem were evident in the style of interactions observed among SMEs and with the *i*AID. The following four interactions styles were observed as follows:

1. All to Artefact (SMEs-*i*AID): All SMEs focused their attention on the single visual display and observed the content of the visual interface (Figure 5a);
2. All to One (SMEs-SME): SMEs focused their attention and listened to one SME who actively expressed a point of view, supported a claim or rejected another SME’s viewpoint and proposed an alternative approach to problem solving (Figure 5b);
3. Pairs, rest to Artifact (SME-SME, SMEs-*i*AID): One SME shared minor observations about the visualization with a nearby SME without engaging the whole group (Figure 5c); and
4. One-on-One (SME-SME): Two SMEs discussed various aspects of the problem solving process, presented their personal perspectives, shared their background knowledge, and convinced each other in a one-on-one setting, before returning to the group conversation (Figure 5d).

### 3.3. Thinking with the Tool

**Figure 6:**
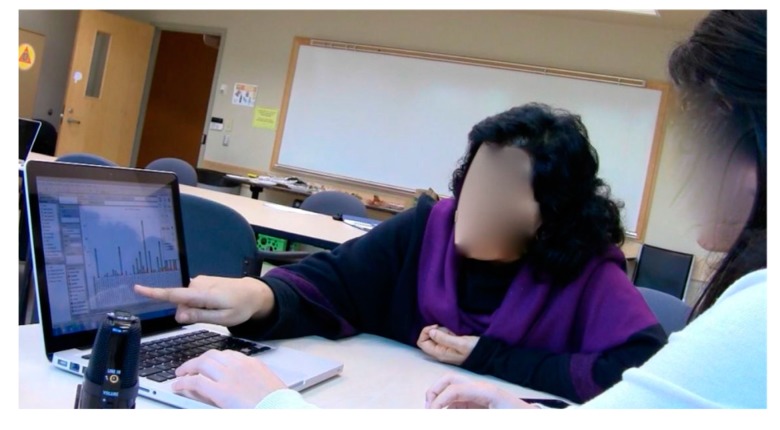
Thinking with the tool.

During the collaborative GA sessions, SMEs manipulated the *i*AID visual display and customized the visualizations according to SMEs’ needs and task requirements. With the assistance of VAE, SMEs interacted with the dashboard to refine the visual representations in a way that enhanced their understanding of the multidimensional injury data and empowered them with the best approach to address the injury problem at hand, raise questions, converge to a solution and reach a consensus (Figure 6). As one SME explained:

SME: *“but look at the ‘Suicide’, low, rise, rise, plateau, and then, but look at that...it’s very interesting! Why, what happened down here, why did it go down?”*

Using a seven point Likert-scale (1—Strongly Agree to 7—Strongly Disagree), SMEs rated the *i*AID dashboard high in terms of “increase learning” (85.6%), “support-problem solving” (100%) and “decision-making processes” (85.6%) (Table 1).

SME expressed their experience interacting with the visualization tool:

- “They (visual representations) provide a great deal of information very quickly and allow me to understand trends and variation in the injury data.”
- “I thought the interaction and having the other experts there as well, people who have used the data differently, I thought that the interaction was really helpful.”
- “It (visual representation) helps to present the story.”
- “I think that our group session was great because we sort of all, everyone got some words in and they were able to work together on that.”
- “It (visual representation) helps to think about the data in different way. It’s kind of different ways to slice the pie to get the creativity going in terms of what the data are actually telling us and possible directions for preventions.”
- “The visual representations of the data for me are key in understanding what I’m looking at.”
- “I trust what I see.”

## 4. Discussion

The Delphi-structured GA sessions incited interactions and exchange of thoughts and ideas among SMEs through conversation and dialogues. This exchange of knowledge served to supplement additional layers of *common ground*, consequently improving the coordination of collaborative activities. In his book ‘Using Language’, Clark explains that according to Thomas Schelling, people coordinate their activities to address coordination problems. He further posits, “Two people have a coordination problem whenever they have common interests, or goals, and each person’s actions depend on the actions of the other” [27]. During the interactive GA sessions, SMEs actively collaborated and engaged with each other to coordinate their joint activities. Their involvement in joint activities revolved around their joint goals, joint intentions, and joint incentive to find a solution to the complex injury problem. The observed patterns of collaboration fell into the following five main collaborative activities within the context of the problem-solving and decision-making process:

1. Collaborate to Explore (C2E): The GA session fostered a dynamic learning environment for SMEs to jointly explore the injury data using the *i*AID and to investigate the health problem in a collaborative social setting.
2. Collaborate to Visualize (C2V): SMEs collaboratively interacted with the shared visual display of the *i*AID dashboard. This visual analytics tool amplified SMEs cognitive capabilities and improved their collaboration in order to facilitate their problem-solving and decision-making process.
3. Collaborate to Argue (C2A): SMEs collaborated to present various viewpoints, to argue broader perspectives and to suggest solutions about the injury problem. During the GA sessions, SMEs communicated their viewpoints with each other, clarified their perspectives, justified their thinking, and supported their claims through dialogue and argumentation.
4. Collaborate to Solve (C2S): SMEs integrated their prior knowledge through interactions and communication to make sense of the problem situation. SMEs collaboratively manipulated the injury data and re-represented the information in an easy-to-understand format to gain insights into the injury data, build knowledge, and solve the analytical task.
5. Collaborate to Decide (C2D): SMEs collaboratively used the *i*AID to monitor the injury situation, assess the current state of the injury problem, and consequently support the decision-making process. SMEs collaboratively embraced an interdisciplinary discussion and argumentation. This process enhanced the chain of inference through the distributed cognition process to solve the analytical problem and reach an informed decision.

SME interactions were tightly linked to their collaborative activities intended to solve the analytical problem. As SMEs attended to a common task and shared mutual goals, they were engaged in collaborative activities facilitated by their various social interactions. SME interactions were pivotal to achieve ‘shared meaning, mutual understanding and the coordination of human conducts’ [29]. One SME explained, “That interaction between all of us, is what I think lead to better problem solving”. Interactions among group members imposed some delay in the problem solving process. However, these interactions empowered SMEs with the ‘assembly effect bonuses’ [30] that synthesized group knowledge and inputs to improve the quality of the analysis outcome. It was evident that the observed interactions between SMEs and with the *i*AID tool in a Delphi structured setting contributed to the multifaceted dimensions of the collaborative analytics process and enabled SMEs to efficiently exchange knowledge and effectively solve the analytical task.

Previous similar studies confirmed current results [19,20,21,31,32,33,34,35,36] and showed that interactions among a group of participants using a visualization aid tool enhances their coordination and collaboration, which in turn improves their problem solving and decision-making processes. In our study, advancing the injury problem-solving process relied heavily on the interactions between SMEs, as well as communication with VAE, to refine and customize the *i*AID visual display, so as to reflect the needs and preferences of SMEs. Turn-taking within the flow of conversation between SMEs was evident, and suggested a joint resolve to solve the injury problem through rounds of argumentation and discussion. Studying these interactions through the lens of language use enabled further understanding of the social and cognitive aspects of the collaborative analytics process. The Delphi structured social group afforded a unique atmosphere of awareness among SMEs to attend to each other’s viewpoints, thus supporting coordinated actions and collaborative activities in order to reach a consensus. In addition to the social collaboration, the perceptual co-presence of the *i*AID influenced joint actions between SMEs. The common visual display not only served to align SMEs discussion and argumentation, it also amplified the cognitive and perceptual capabilities of SMEs to effectively understand and interpret the injury data. SMEs discussed, exchanged knowledge, and reasoned about the injury problem using the visual analytics dashboard. The *i*AID visual display guided stakeholders to move forward based on the gained knowledge from the shared visual representation. Attending to the same visual representation made it easier for SMEs to refer to the data, to reflect on the generated visualizations, to communicate their viewpoints and ideas, and consequently to advance the problem-solving process.

**Figure 7:**
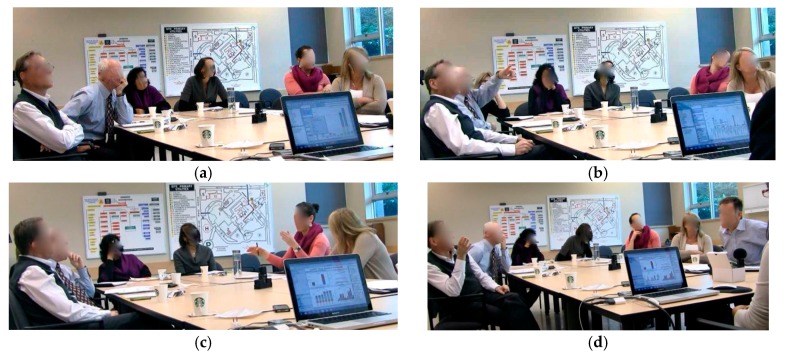
(**a**) Body language; (**b**) tool gestures; (**c**) hand gesture; and (**d**) body posture.

Off-screen communication was observed between SMEs by way of the gestures captured on video. Gestures constitute an essential component of the conversational interactions among individuals [37]. Gestures are closely associated with speech to support the expression of thoughts and ideas throughout the interactive communication process [38]. Throughout the collaborative analytics sessions, hand gestures and body language (Figure 7a,c,d) were essential to help SMEs interpret and direct communicative activities. We parsed the video sessions and coded various SMEs’ gestures that contributed to the collaborative group analytics phenomena. We noticed that throughout the sessions, SMEs finger-pointed to refer to the visualization as a focal point and to orient SMEs attention to a particular space on the screen. These hand gestures were mainly “tool gestures” (Figure 7b). The tool gestures were essential to guide SMEs’ cognitive attention to a specific visual display on the dashboard in order to either gain insights into the injury data or to support SMEs’ viewpoints with evidence from the visualization. SMEs also gestured with their hands to explain a new point of view or to show patterns in the data (Figure 7c). Other gestures, like head nodding, were interlinked with activities related to discussion and argumentation, they revealed SMEs’ understanding and approval of the declared statement. Body posture (Figure 7d) showed SMEs’ interest to attend to an individual SME viewpoint. These gestures played a substantial role in facilitating the communication of thoughts and ideas among SMEs, their coordination of activities and, consequently, advancement of the problem solving process. 

The combination of cognitive efforts from multiple SMEs, facilitated and supported by the surrounding computer-mediated environment, enhanced individuals” abilities to perceive and reason complex cognitive tasks [39]. The *i*AID acted as a coordination device [28] to support SMEs’ analytical processes. Integrating this coordination device throughout the Delphi-structured GA sessions served to offload SMEs’ cognitive processes to more interactions with the dashboard. The process of exchange and transfer of knowledge through the manipulation and refinement of the visualization tool during the interactive GA sessions helped to convey information that was essential for SMEs to build cumulative knowledge and understanding about the injury data otherwise not easily retrieved from the final visualization. Interactions with other group members, interactions with the *i*AID dashboard, as well as interaction with the social and cultural environment, represented key components of the cognitive and computational system that supported SMEs’ analytical problem solving process and decision-making.

We acknowledge that our GA study encounters a number of limitations. Firstly, we had a relatively small sample size, which may limit the generalizability of the study and its applicability to the larger injury stakeholders’ population and the broader public health community. This restriction is imposed by patient information privacy policies that allow only a limited number of authorized stakeholders to access and interact with the available public health data. Secondly, this GA study investigated a hypothetical injury issue and therefore injury stakeholders were not making actual decisions. However, it was essential to acknowledge and take into consideration the challenges and obstacles that might face the collaborative decision-making process, during the course of the group analysis session. Thirdly, injury stakeholders were manipulating the dashboard visual display with the assistance of the VAE during the pilot GA sessions. To help injury practitioners and policy-makers independently generate visualization and accurately analyze data, structured and guided eLearning tutorials should be offered to novice users to acquaint them with the basic concepts and the essential functionalities of the dashboard. ‘Know-how’ or a ‘help’ feature can be integrated into the dashboard to help teach novice users about the dashboard features, provide support on a need-basis and suggest appropriate visualizations based on the type of data analyzed.

Following this pilot study, a complementary research work was carried out to conduct an extensive beta testing sessions for dashboard users [40]. The study served to understand users experience working independently on the dashboard, without VAE assistance and to gather feedback that was then used to refine the tool and make the dashboard more intuitive and easy to use by injury practitioners and policy makers outside of the group analytics setting.

Despite these limitations, the study findings clearly reveal the advantages of integrating the analytical dashboard as a decision-support tool to synthesize information from multidimensional and dynamic health data. The study’s empirical findings constitute relevant and valuable resources that contribute to the understanding of collaborative visual analytics and its impact on problem-solving and decision-making within the health care sector and, specifically, to injury prevention. This pilot study of the GA methodology has many implications for future research. The results may inform future innovative visualization tools that will synthesize key social and collaborative components of visual analytics to enhance group knowledge construction and optimize decision-making. Furthermore, GA methodology can be applied to other domains within the healthcare sector which would likely benefit from interdisciplinary insights [41]. Examples may include analysis of trauma registries or emergency department data so as to generate new evidence based knowledge and address the needs of these populations. The next phase of the present study constitutes the design and evaluation of an online dashboard expanded to include injury data from across Canada. This national dashboard can then be integrated into the national public health web portal and used to assist Canadian health professionals and policy-makers in making informed and timely decisions regarding the most appropriate actions to improve child and youth injury prevention efforts in Canada.

## 5. Conclusions

Developing effective interventions to prevent injuries is a challenging task, as injury itself is considered a complex health problem due to its multidimensional nature. To address such challenges inherent in interpreting complex data, we proposed a collaborative method—group analytics (GA), supported by an interactive visualization tool, and theoretically grounded in Clark’s JAT. This pilot study successfully demonstrated the practical application of group analytics in injury prevention to facilitate data analysis, problem-solving, and consensus decision-making for multiple injury stakeholders. The results of the analysis showed that the group analytics methodology empirically evaluated and assessed the impact of collaborative visual analytics on problem-solving and decision-making processes within the context of public health wicked problems. The analytical sessions helped us to successfully capture stakeholders’ reasoning process in a real-world setting. The integration of an interactive visualization tool proved to afford injury stakeholders a useful platform from which to investigate the injury data, effectively discuss retrieved information, and share perspectives related to the analytical injury problem. Pooling multiple injury stakeholders’ ideas and inputs fostered a collective intelligence environment supported by the use of the designed *i*AID dashboard, helped to address and solve the analytical injury problem. Engaging with the right stakeholders in a real-world setting presented an exceptional opportunity to assess the effect of collaborative visual analytics on addressing a complex health problem. Future work can expand the presented group analytics methodology to other domains of public health, in order to facilitate interdisciplinary analysis of complex data, and ultimately improve the capacity of stakeholders to make timely, evidence-informed decisions. 

## Figures and Tables

**Table 1:** SMEs rating their interactions with the tool.

Variables	Strongly Agree	Agree	Somehow Agree	Neutral	Somehow Disagree	Disagree	Strongly Disagree
1. Increase Learning	57.1%	28.5%	14.4%	0%	0%	0%	0%
2. Support Problem-Solving	57.1%	42.9%	0%	0%	0%	0%	0%
3. Support Decision-Making	57.1%	28.5%	0%	14.4%	0%	0%	0%

## Appendix A

### Appendix A.1. CHIRPP Data

We used the Canadian Hospitals Injury Reporting and Prevention Program (CHIRPP) database to retrieve data for this research study [12]. CHIRPP is a computerized information system that collects and analyzes data on injuries to people, mainly children, who are seen at the emergency rooms of 10 paediatric hospitals and of four general hospitals in Canada. In preparation for the analytical sessions, the CHIRPP dataset was cleaned and arranged to support the analysis process and match the requirements of the proposed task. We ensured the inclusion of fields that were relevant to the analytical task such as injury type, injury causes, injury sub-causes, injury location, injury intent, as well as age group and gender. Prior to the group analytics sessions, the CHIRPP data was prepared and loaded into the designed *i*AID dashboard. In addition to the traditional data cleaning process, a decision was made regarding the selection of relevant dimensions and data variables that needed to be connected to the *i*AID dashboard in order to support SMEs in completing their analytical task. These variables included injury causes, types and intents, injured people sex and age group, as well as injury locations.

### Appendix A.2. Interactive Analytical Injury Dashboard (iAID)

The main purpose of the *i*AID dashboard is to help injury stakeholders understand complex and heterogeneous injury data and effectively solve real-world injury problems. Typically, a dashboard is limited to the visual display of key indicators [13]. In addition to the visualization of key indicators, we aimed to design a dashboard that integrated stakeholders’ analytical processes and supported their reasoning through the use of interactive interfaces and customized visualizations in coordinated views [4]. The visualizations were selected to depict relevant aspects of the injury problem and assist injury stakeholders or SMEs in building knowledge and making informed decisions about dynamic situations for child and youth injury prevention initiatives.

Tableau Software was used to design and build the main visualizations of the *i*AID dashboard. Specific objectives of the *i*AID dashboard included: (i) it should offer rapid synthesis and interpretation of complex and multidimensional injury data; and (ii) it should support injury stakeholders’ analytical and cognitive tasks in order to facilitate their problem solving and support decision-making processes.

The *i*AID dashboard offers injury stakeholders summative information of key injury indicators in an intuitive interface, easy to use tool and easy to interpret visualizations. The dashboard provides the following main functionalities:

- *i*AID built an overview of the injury situation and the injury indicators trending over time and across different health authorities (HA) and health service delivery areas (HSDA), answering users ‘When?’ and ‘Where?’ questions (Figure 1).
- *i*AID offered drill-down capabilities for additional levels of granularity (Figure 2).
- *i*AID offered interactive filtering for stakeholders to be able to partition the data and focus on a specific interesting subset of the injury indicators data (Figure 1).
- *i*AID offered interactive zooming using the provincial map of British Columbia where the injury indicators data are compressed in the form of a pie chart to show an overview of the indicators’ distribution across various health authorities (Figure 1).
- *i*AID offered interactive distortion to show areas of interests with high level of details while keeping other indicators displayed with low level of detail (Figure 2).
- *i*AID offered details-on-demand to allows users to interactively select parts of data to be visualized in more details while providing an overview of the whole informational concept. Users can hover the mouse over a specific area of the visualizations and obtain details-on-demand information (Figure 3).

*i*AID offered interactive linking and brushing to combine different visualization methods to overcome the shortcomings of single techniques. Brushing means selecting a subset of the injury indicators data, by highlighting it with a mouse click, and the linking technique will enable stakeholders to see how this particular data subset behaves in each of the visualizations in different windows of the dashboard (Figure 3).
